# Therapeutic hexapeptide (PGPIPN) prevents and cures alcoholic fatty liver disease by affecting the expressions of genes related with lipid metabolism and oxidative stress

**DOI:** 10.18632/oncotarget.21404

**Published:** 2017-09-30

**Authors:** Nan Qi, Chen Liu, Haoran Yang, Wanrong Shi, Shenyi Wang, Yan Zhou, Cai Wei, Fang Gu, Yide Qin

**Affiliations:** ^1^ Department of Biochemistry and Molecular Biology, School of Basic Medical Sciences, Anhui Medical University, Hefei, Anhui 230032, China; ^2^ Clinical Laboratory, Guangming Center Hospital, Shenzhen, Guangdong 518107, China; ^3^ Center of Medical Physics and Technology, Hefei Institutes of Physical Science, Chinese Academy of Sciences, Hefei, Anhui 230031, China; ^4^ Department of Internal Medicine, The Fourth Affiliated Hospital of Anhui Medical University, Hefei, Anhui 230022, China; ^5^ Department of Pharmacy, The Second Affiliated Hospital of Anhui Medical University, Hefei, Anhui 230601, China

**Keywords:** PGPIPN, alcoholic fatty liver, steatosis, gene expression

## Abstract

PGPIPN is a therapeutic hexapeptide derived from bovine β-casein. Here we investigated the role and mechanism of this peptide on alcoholic fatty liver disease (AFLD). We took human hepatic cell line LO2 and hepatocellular carcinoma cell line HepG2 to establish the models of steatosis hepatocyte induced by alcohol, taken PGPIPN as pharmacological intervention. And we also established the model of AFLD mice, taken PGPIPN as therapeutic drug and glutathione (GSH) as positive control. We assayed the biochemical materials related to liver injury, lipid metabolism and oxidation, and observed morphology change and fat accumulation of hepatocyte. The gene expressions and/or activities related to liver injury, lipid metabolism and oxidation, such as ACC, PPAR-γ, CHOP and Caspase-3, were assessed by real time PCR and western blot. Our results showed PGPIPN alleviated hepatic steatosis in both model cells and AFLD model mice. PGPIPN can effectively reduce the lipid accumulation and oxidative stress of hepatocyte in a dose-dependent manner. PGPIPN alleviated alcohol-induced cell steatosis and injuries by regulating the gene expressions and/or activities of ACC, PPAR-γ, CHOP and Caspase-3. Our results demonstrated PGPIPN had the protective and therapeutic effect on AFLD, which may serve as a potential therapeutic agent for AFLD.

## INTRODUCTION

Alcoholic liver disease encompasses a spectrum of jury, ranging from alcoholic fatty liver disease (AFLD) or simple steatosis to cirrhosis, which can further progress to hepatocellular carcinoma [1]. The amount of alcohol ingested is the most important risk factor for the development of alcoholic liver diseases [2]. The conventional course of therapy for alcoholic liver disease includes steroid and anti-cytokine therapy [3]. Although much progress has been achieved in the development of alcoholic liver disease therapy in recent years, problems continue to arise due to their side-effects [4]. It is crucial to treat alcoholic steatosis during the early stage of AFLD and prevent the progression to more severe forms of liver damage. Therefore, there is a need to develop safe and effective agents against AFLD.

Bioactive peptide therapeutics is an immerging area for human disease. The peptides are easily obtained either from nature resources such as foods or artificially design based on the target protein structure. A substantial portion of biologically active peptides are the cryptic peptides, which are short sequences of proteins available all over the world. When proteins are hydrolyzed, such as digestive enzymes, these peptides will be released. The bioactive peptides are generally used as functional food ingredients, nutricosmeceuticals or therapeutic agents [5]. These are nearly hundred formally authorised peptides and several hundred peptides currently undergoing preclinical and clinical development, these interesting molecules have conquered their place in the pharmacological field [6].

Therefore, significant efforts have been undertaken recently to investigate the biological functionalities of some bioactive peptides including antioxidative, antihypertensive, antidiabetic, anticancer and immunomodulatory activities [7, 8]. Recent studies have shown that bioactive peptides have protective effects on alcohol-induced liver damage [9]. These studies also show that some peptides or cytokines have the ability to accelerate alcohol clearance, and significantly to lower serum levels or activities of total cholesterol (TC), triglyceride (TG), alanine aminotransferase (ALT) and asparatate aminotransferase (AST) [9].

Milk-derived bioactive peptides can be encrypted in both casein and whey proteins [10]. These peptides are inactive within the protein sequence, requiring enzymatic proteolysis for release of the bioactive fragment from the proteins precursor [11]. PGPIPN (Pro-Gly-Pro-Ile-Pro-Asn, residues 63–68 of bovine β-casein) was found to have immunomodulatory function *in vivo*. Generally, short peptide (usually less than or equal to 7 amino acids) can be directly absorbed into the blood through the digestive tract [5]. The low bioavailability of many orally administered peptides results from being degraded by digestive enzymes in the gastrointestinal tract [12]. However, this peptide containing three prolines can resist the hydrolysis of proteaseto [13]. In the pre-experiments, we also used liquid chromatography−mass spectrometry (LC-MS) to detect this peptide in mice blood at (83.58±10.16) ng/ml in 1 hour after the mice were gavaged PGPIPN at 250μg/kg body weight (data not shown).

Previous studies have shown that PGPIPN could play an important role in immune response in rats and mice, such as promoting macrophage phagocytosis and lymphocyte proliferation [14]. Moreover, our subsequent studies demonstrated that PGPIPN also had good antioxidant effect *in vivo* (data not shown). This intrigues us to investigate whether PGPIPN can protect against alcohol-induced liver jury.

The findings in the present study provide the proof of concept for using PGPIPN as a potential therapeutic agent for the treatment of AFLD.

## RESULTS

### PGPIPN alleviated alcohol-induced cell steatosis and injuries in human liver cell line LO2 and hepatocellular carcinoma cell line HepG2

**Figure 1:**
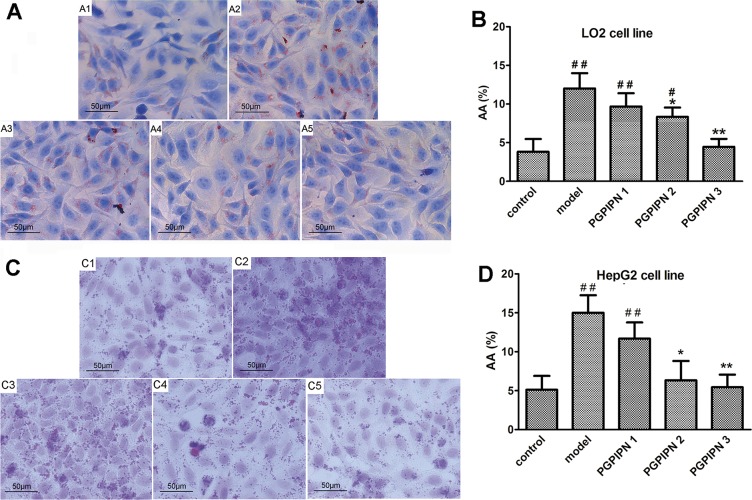
PGPIPN alleviated alcohol-induced cell steatosis in both LO2 and HepG2 cells **(A)** and **(C)** LO2 and HepG2 cells stained with Oil Red O were respectively observed under high power microscope (Oil Red O stained, ×400), (A1 and C1) control group, (A2 and C2) model group induced by alcohol, (A3 and C3) PGPIPN 1 group treaded with 0.15μmol/L PGPIPN, (A4 and C4) PGPIPN 2 group treaded with 1.5μmol/L PGPIPN, (A5 and C5) PGPIPN 3 group treaded with 15μmol/L PGPIPN. **(B)** and **(D)** The LO2 and HepG2 cells stained with Oil Red O were respectively analyzed with Image-Pro Plus 7.0 image analysis software. Positive area in measurement area, AA (%) =positive area/total area×100%. The data are shown as means ± SD of three independent experiments, ^*^P<0.05, ^**^P<0.01 compared with model,^#^*P*<0.05, ^##^*P*<0.01 compared with control.

We employed human liver cell line LO2 and hepatocellular carcinoma cell line HepG2 as the models to study AFLD *in vitro*. The lipid loading in cells was observed by staining the lipid droplets with Oil Red O. Compared with control groups (Figure 1A1 and 1C1), the alcohol-induced LO2 and HepG2 cells in model groups revealed conspicuous steatosis (Figure 1A2 and 1C2), in which LO2 and HepG2 cells were engorged with lipid droplets. However, we observed that lipid-droplets in LO2 and HepG2 cells were significantly reduced in PGPIPN 1, 2 and 3 (Figure 1A3, 1A4, 1A5, 1C3, 1C4 and 1C5) groups, PGPIPN treatment took effect in a dose-dependent manner (Figure 1B and 1D).

The morphologies and changes of liver cell line LO2 treaded with alcohol and/or peptide were observed by transmission electron microscope, as shown in Supplementary Figure 1. Compared with control group, LO2 cells in model group showed mitochondrial swelling accompanying disappearance of cristae and particles shedding from rough endoplasmic reticulum (RER). PGPIPN intervention alleviated the damage and stress of liver cell mitochondria and RER.

**Figure 2:**
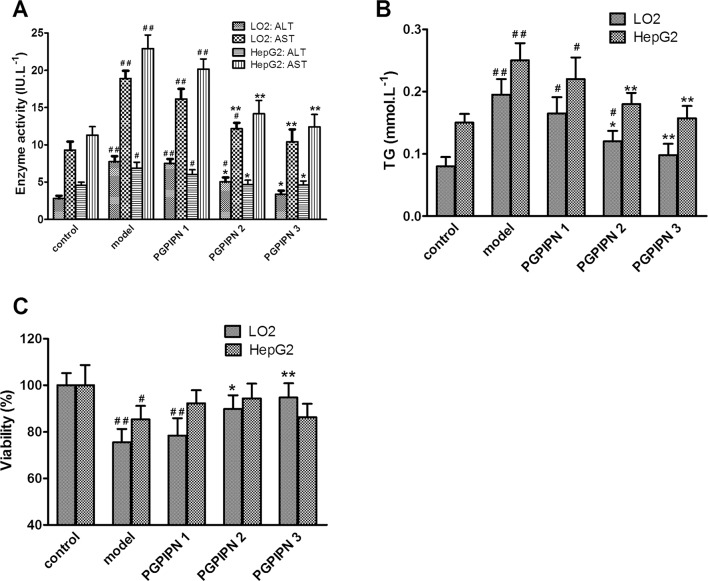
PGPIPN decreased the levels of ALT and AST leaking in the culture medium and intracellular triglyceride (TG), and affected the cell viabilities in both LO2 and HepG2 cells induced by alcohol *in vitro* **(A)** The levels of ALT and AST leaking in the culture medium. **(B)** The intracellular TG concentrations in cells. **(C)** The viabilities of cells. PGPIPN 1, 2 and 3 were treaded with 0.15, 1.5 and 15μmol/L PGPIPN, respectively. The data shown are mean ± SD of three independent experiments, ^*^P<0.05, ^**^P<0.01 compared with model, ^#^*P*<0.05, ^##^*P*<0.01 compared with control.

Compared with the control groups, the activities of ALT and AST leaking in the culture medium in model groups increased significantly. However, PGPIPN intervention significantly reduced the ALT and AST activities, see Figure 2A. Biochemical assays revealed higher intracellular TG in the model groups compared with control groups, while PGPIPN treatment could decrease TG levels (Figure 2B).

### PGPIPN increased the viabilities of human normal hepatic cell line LO2 cells treated with alcohol, but had little effect on that of hepatocellular carcinoma cell line HepG2 cells

PGPIPN intervention could significantly alleviate the inhibitory effect of ethanol on the viabilities of LO2 cells (Figure 2C). Compared with model group, the viabilities of LO2 cells in PGPIPN 2 and 3 groups were significantly enhanced. The viability in PGPIPN 3 group was close to the control group. However, PGPIPN had little effect on the viabilities of HepG2 cells. Compared with the model group, PGPIPN caused no significant increase in the viabilities of HepG2 cells (Figure 2C).

### PGPIPN prevented and attenuated AFLD of model animals *in vivo*

**Figure 3:**
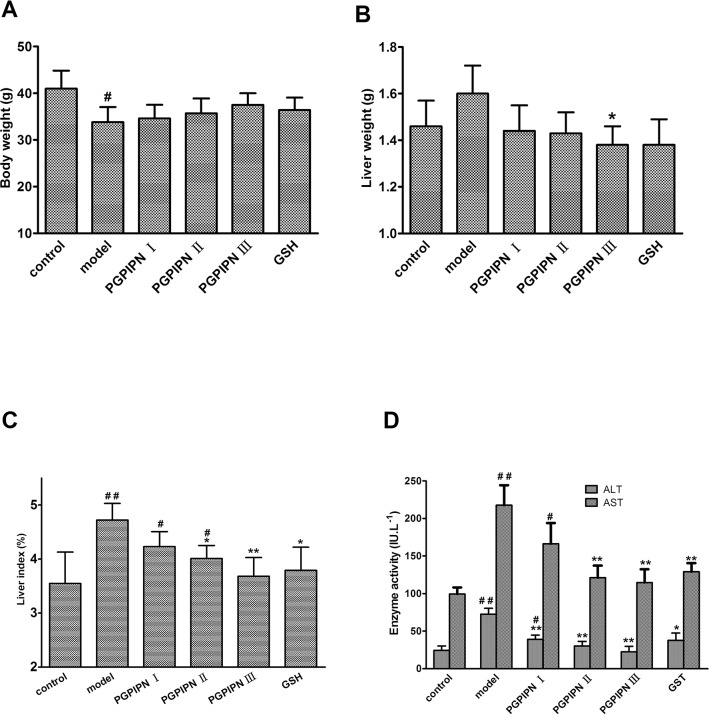
PGPIPN attenuated liver index and the levels of serum ALT and AST in model mice *in vivo* **(A)** Body weight in model mice. **(B)** Liver weight in model mice. **(C)** Liver index in model mice. **(D)** The levels of serum ALT and AST in model mice. The data are shown as means ± SD of 10 mice in each group, ^*^P<0.05, ^**^P<0.01 compared with model, ^#^*P*<0.05, ^##^*P*<0.01 compared with control.

To determine whether PGPIPN could prevent and remedy AFLD *in vivo*, sixty healthy male Kunming mice were divided into six groups (control, model, PGPIPN I, PGPIPN II, PGPIPN III and GSH (glutathione)) with 10 mice in each group as described in Materials and Methods. We successfully built AFLD animal models in model group with 10 mice. Compared with the control group, the mice in model group had a lighter body weight (Figure 3A), but the liver weight increased (Figure 3B). PGPIPN intervention could obviously improve the mice healthy, attenuate above symptoms displaying in model group. As shown in Figure 3C, compared with the control group, liver index of mice in the model group was significantly higher, and PGPIPN intervention could reduce liver index of mice in which the differences reached significance in PGPIPN II and III groups. Compared with the control group, the ALT and AST activities in serum of mice in model group increased significantly, and PGPIPN intervention significantly reduced their activities, see Figure 3D.

**Figure 4:**
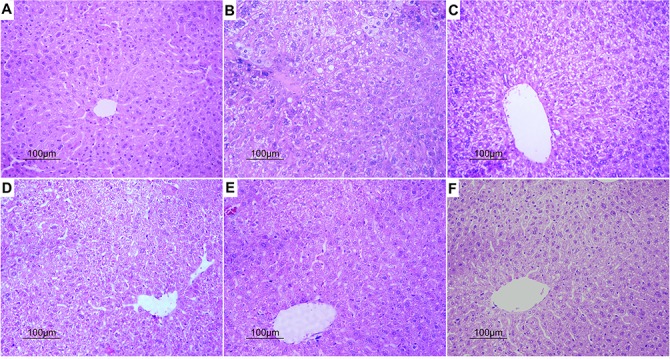
PGPIPN attenuated pathological changes of hepatocyte in model animals induced with alcohol-intake *in vivo* (H&E stained, ×200) **(A)** Control group. **(B)** Model group. **(C)** PGPIPN I group. **(D)** PGPIPN II group. **(E)** PGPIPN III group. **(F)** GSH group.

**Figure 5:**
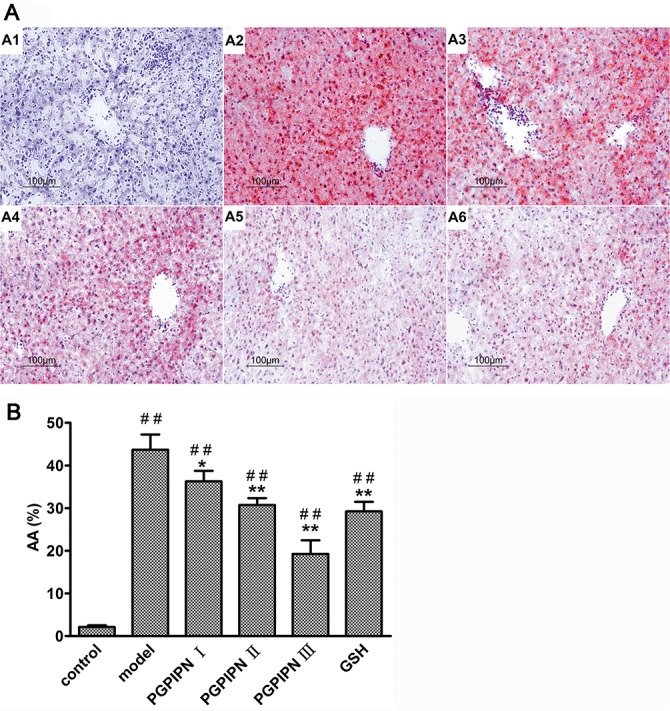
PGPIPN attenuated hepatocyte steatosis of model animals induced with alcohol-intake *in vivo* **(A)** The liver tissues stained with Oil Red O were observed under high power microscope (Oil Red O stained, ×200), (A1) control group, (A2) model group, (A3) PGPIPN I group, (A4) PGPIPN II group, (A5) PGPIPN III group, (A6) GSH group. **(B)** The Oil Red O staining tissue sections were analyzed with Image-Pro Plus 7.0 image analysis software. Positive area in measurement area, AA (%) =positive area/total area×100%. The data are shown as means ± SD of 10 mice in each group, ^*^P<0.05, ^**^P<0.01 compared with model, ^##^*P*<0.01 compared with control.

Based on hematoxylin and eosin (H&E) staining histological analysis, severe pathological changes (steatosis) were detected in model group mice (Figure 4), as shown by the disarranged liver structure as well as the increased number of vacuoles. PGPIPN treatment significantly alleviated steatosis in PGPIPN I, II and III group mice (Figure 4). The alcohol gavage dramatically increased lipid deposition in pericentral areas, as manifested by the accumulation of Oil Red O-positive lipid droplets in model group mice (Figure 5). PGPIPN treatment significantly diminished fat accumulations of mice livers in PGPIPN I, II and III groups in a dose-dependent manner (Figure 5 and Table 1).

**Table 1:** PGPIPN attenuated hepatocyte steatosis of model animals induced with alcohol-intake *in vivo* by observing Oil Red O staining specimens under high power microscope

Group (n=10)	Hepatic steatosis area	Hepatic steatosis degree
control	<10%	0 (normal)
Model	>66%	4 (severe fatty liver)
PGPIPN I	51~66%	3 (moderate fatty liver)
PGPIPN II	34~50%	2 (mild fatty liver)
PGPIPN III	11~33%	1 (slight fatty liver)
GSH	34~50%	2 (mild fatty liver)

### PGPIPN attenuated alcohol-intake induced hepatic lipid metabolic disturbance and oxidative stress of model animals *in vivo*

**Figure 6:**
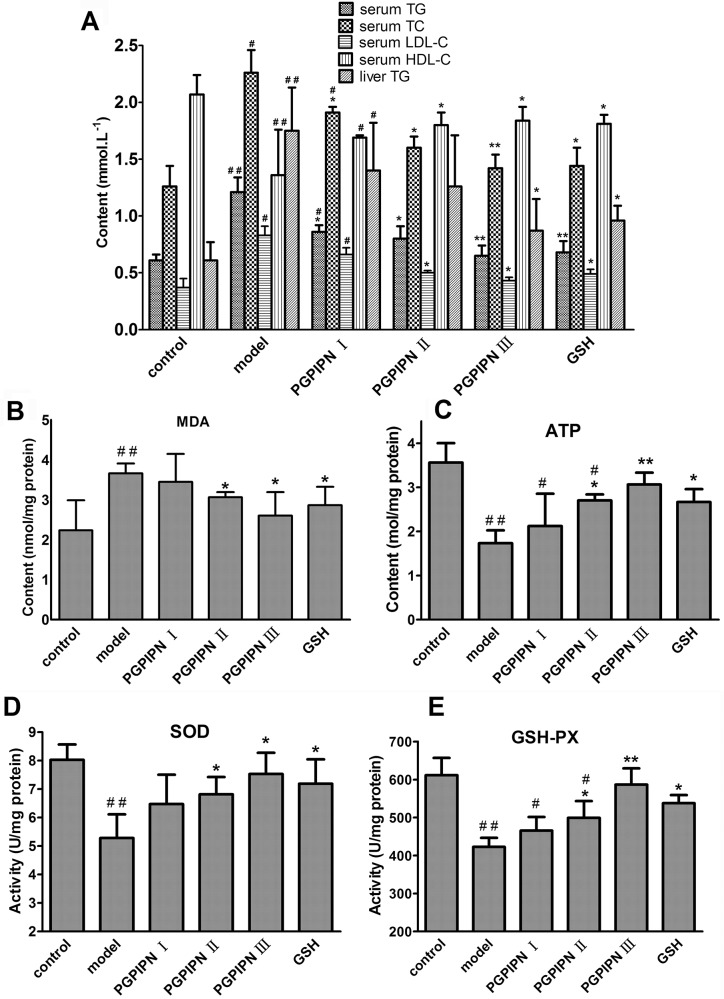
PGPIPN attenuated hepatic lipid metabolic disturbance and oxidative stress of model animals induced with alcohol-intake *in vivo* **(A)** Some biochemical materials related to liver lipid metabolism in the serums and liver tissues (TG: total triglyceride, TC: total cholesterol, LDL-C: low density lipoprotein-cholesterol, HDL-C: high density lipoprotein-cholesterol). **(B)** Malondialdehyde (MDA) in the liver tissues. **(C)** Adenosine triphosphate (ATP) in the liver tissues. **(D)** Superoxide dismutase (SOD) in the liver tissues. **(E)** Glutathione peroxidase (GSH-PX) in the liver tissues. The data are shown as means ± SD of 10 mice in each group, ^*^P<0.05, ^**^P<0.01 compared with model, ^#^*P*<0.05, ^##^*P*<0.01 compared with control. Note: The above biochemical materials in liver are these concentrations in liver homogenate of 10% w/v (the fresh liver tissue / saline).

Biochemical assays revealed higher serum TG, TC and LDL-C (low density lipoprotein-cholesterol) and liver TG (TG concentrations in liver homogenate) in the model group mice compared with control group, while serum HDL-C (high density lipoprotein-cholesterol) was lower than that in model group (Figure 6A). However, PGPIPN treatment could rectify these changes, including decreasing TG, TC and LDL-C levels and increasing HDL-C level (Figure 6A). This shows that PGPIPN could reduce metabolic disturbance through the regulation of lipid metabolism.

Alcohol gavage significantly induced oxidative stress, as evidenced by increasing hepatic malondialdehyde (MDA) level, and decreasing glutathione peroxidase (GSH-PX) and superoxide dismutase (SOD) activities and adenosine triphosphate (ATP) content in the model group (Figure 6B, 6C, 6D and 6E). PGPIPN effectively reduced the hepatic MDA, and promoted GSH-PX and SOD activities and ATP contents of mice liver homogenates in PGPIPN I, II and III groups (Figure 6B, 6C, 6D and 6E).

### PGPIPN regulated the mRNAs of *ACC*, *PPAR*-γ, *CHOP* and *caspase*-3 genes related with lipid metabolism and oxidative stress in hepatic cells

**Figure 7:**
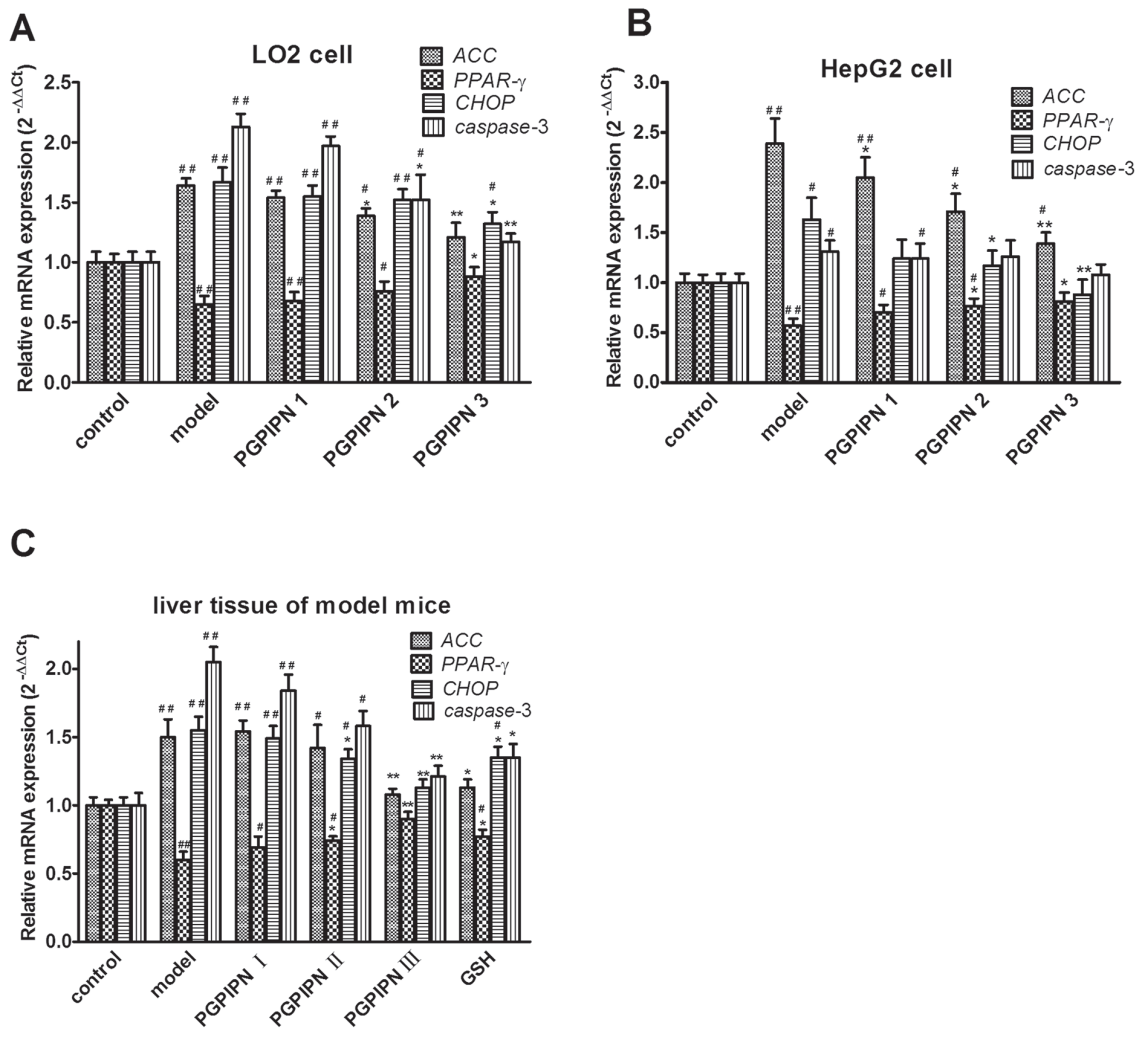
PGPIPN regulated the mRNAs of ACC, PPAR-γ, CHOP and caspase-3 genes related with lipid metabolism and oxidative stress in hepatic cells by real-time PCR RNAs were respectively harvested from **(A)** LO2 and **(B)** HepG2 cells in different groups; mRNAs of *ACC*, *PPAR*-γ, *CHOP* and *caspase*-3 genes were detected by real-time PCR (n=9). **(C)** RNAs were harvested from liver tissues of model mice induced with alcohol-intake in different groups; mRNAs of *ACC*, *PPAR*-γ, *CHOP* and *caspase*-3 were measured by real-time PCR (n=10). The data in A, B and C are shown as means ± SD,^*^P<0.05, ^**^P<0.01 compared with model, ^#^*P*<0.05, ^##^*P*<0.01 compared with control, taken β-*actin* as reference gene.

To explore the potential mechanism of which PGPIPN ameliorated alcohol-induced hepatic steatosis, the expressions of some metabolism-related and oxidative stress genes were examined by real time PCR. Compared with control cells, the mRNAs of *ACC* (acetyl-CoA carboxylase), *CHOP* (CCAAT/enhancer-binding protein homologous protein) and *caspase*-3 significantly increased and *PPAR*-γ (peroxisome proliferator activated receptor γ) mRNAs significantly decreased in the alcohol-induced model groups of human normal liver cell line LO2 (Figure 7A), hepatocellular carcinoma cell line HepG2 (Figure 7B) and model animal (Figure 7C). PGPIPN significantly decreased the mRNA levels of *ACC* and *CHOP* genes, and increased that of *PPAR*-γ gene in contrast. About *caspase*-3 gene, PGPIPN also significantly decreased its mRNA levels in both human liver cell line LO2 (Figure 7A) and model animal (Figure 7C), but did not cause significant changes in HepG2 cells (Figure 7B). The effect of PGPIPN on mRNA expression levels of *ACC*, *PPAR*-γ, *CHOP* and *caspase*-3 (except for HepG2 cells) genes were dose-dependent.

### PGPIPN regulated contents and/or activities of ACC, PPAR-γ, CHOP and Caspase-3 proteins related with lipid metabolism and oxidative stress in hepatic cells

**Figure 8:**
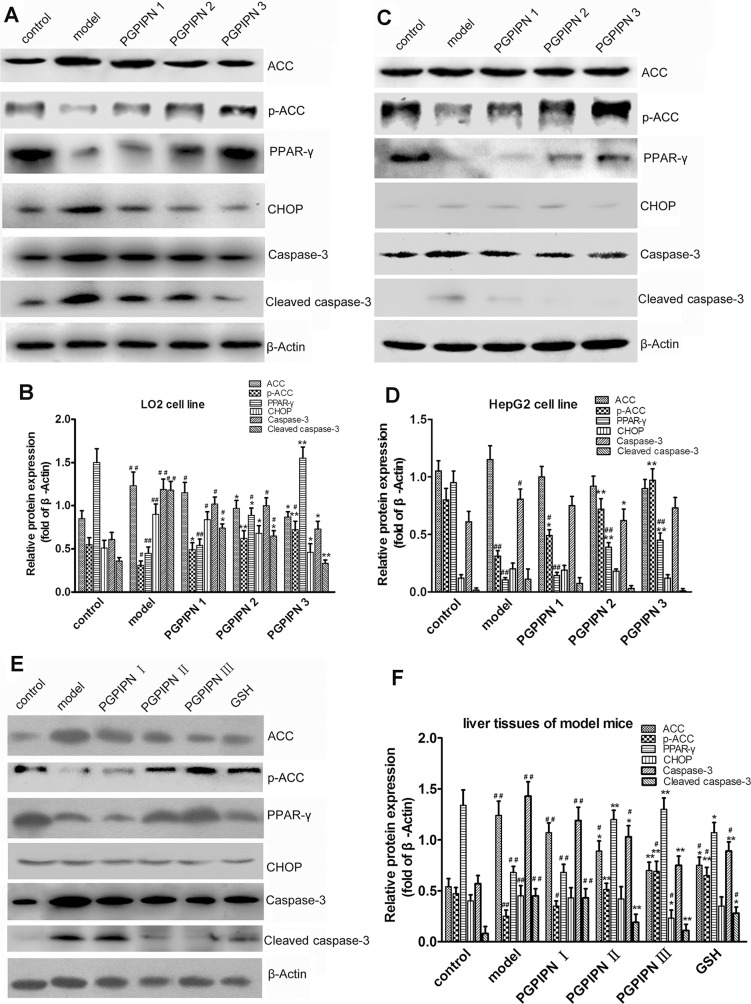
PGPIPN regulated contents and/or activities of ACC, PPAR-γ, CHOP and Caspase-3 proteins related with lipid metabolism and oxidative stress in hepatic cells Western blot analysis was respectively performed in **(A)** LO2 and **(C)** HepG2 cells of different groups and antibodies specific to ACC, pACC, PPAR-γ, CHOP, Caspase-3 and Cleaved caspase-3 were used to assess protein levels. The β-Actin was used to show the similar amount of protein loaded in different lanes. The relative intensities of protein bands in **(B)** A and **(D)** C were determined using Quantity-One software and normalized using β-Actin band intensity (n=9). **(E)** ACC, pACC, PPAR-γ, CHOP, Caspase-3 and Cleaved caspase-3 in liver tissues of model mice induced with alcohol-intake in different groups were detected with western blot analysis and antibodies specific to ACC, pACC, PPAR-γ, CHOP, Caspase-3 and Cleaved caspase-3. The β-Actin was used to show the similar amount of protein loaded in different lanes. **(F)** The relative intensities of protein bands in E were determined using Quantity-One software and normalized using β-Actin band intensity (n=10). The data in B, D and F are shown as means ± SD, ^*^P<0.05, ^**^P<0.01 compared with model, ^#^*P*<0.05, ^##^*P*<0.01 compared with control.

Western blotting was used to analyze ACC, pACC (phosphorylated ACC), PPAR-γ, CHOP, Caspase-3 and Cleaved caspase-3 proteins in both model cells and model animals (Figure 8). In LO2 cells, compared with control group, the alcohol-induced LO2 cells in model group revealed that ACC, CHOP, Caspase-3 and Cleaved caspase-3 significantly increased, and pACC and PPAR-γ significantly decreased in contrast (Figure 8A and 8B). However, PGPIPN treatment significantly diminished these changes in PGPIPN 1, 2 and 3 groups (Figure 8A and 8B). In particular, PGPIPN promoted phosphorylation of ACC and restrained conversion of Caspase-3 to Cleaved caspase-3, thereby inactivated them (Figure 8A and 8B). In HepG2 cells, change patterns of pACC and PPAR-γ were the same as these in LO2 cells. However, ACC and Caspase-3 changed little in HepG2 cells. Except for the slight increase of Cleaved caspase-3 in model group, the contents of CHOP and Cleaved caspase-3 were very low and varied little (Figure 8C and 8D). PGPIPN also affected ACC, pACC, PPAR-γ, CHOP, Caspase-3 and Cleaved caspase-3 proteins in model animal liver cells, which were similar to that of LO2 cells (Figure 8E and 8F). In total, PGPIPN effects were notable in model animal, except in PGPIPN I group at low dose (0.025mg/kg body).

**Figure 9:**
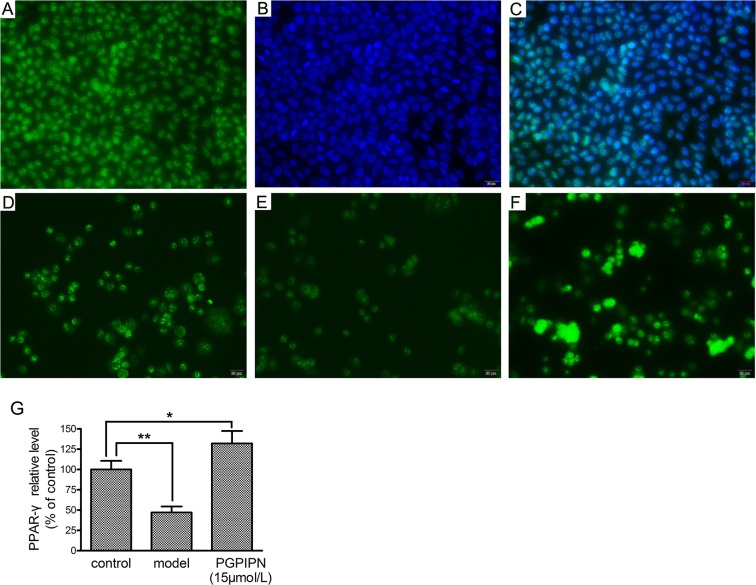
PPAR-γ (peroxisome proliferator-activated receptor gamma) located in the nucleus and PGPIPN up-regulated PPAR-γ level in human hepatocyte line LO2 cells (×400) **(A)** LO2 cells stained with anti-PPAR-γ-FITC. **(B)** LO2 cells stained with nuclear dye DAPI. **(C)** The merge of A and B pictures. **(D)** Control group stained with anti-PPAR-γ-FITC. **(E)** Model group stained with anti- PPAR-γ-FITC. **(F)** PGPIPN treaded group (with 15μmol/L PGPIPN) stained with anti- PPAR-γ-FITC. **(G)** The relative quantitative changes in the PPAR-γ expression (n=9). The data in G are shown as means ± SD, ^*^P<0.05, ^**^P<0.01 compared with control.

Immunofluorescence staining in LO2 cells displayed that PPAR-γ all located in the nucleus and PGPIPN up-regulated PPAR-γ level (Figure 9), as the same as that in western blotting.

## DISCUSSION

PGPIPN from bovine milk protein has demonstrated various effects including immunoregulation and anti-cancer [15, 16]. The milk peptides are safe to consume orally for healthy volunteers and no clinically significant side effects are reported [17]. In this study, we provided compelling evidence for a unique role of PGPIPN in alleviating chronic alcohol intake-induced hepatic steatosis both *in vitro* and *in vivo*, suggesting that PGPIPN has a significant protective effect for liver against AFLD.

In the initial stage of AFLD, TG accumulates in hepatocytes leading to the development of steatosis, which is a reversible condition [18]. The pathogenesis of AFLD includes oxidative stress and inflammatory response [19–21], which may lead to liver injury and the leakage of ALT and AST from damaged hepatocytes. Increase reactive oxygen species (ROS) can promote lipid peroxidation. Previous studies showed MDA, SOD and GSH-PX played vital roles in ROS-mediated lipid peroxidation and liver injury [22–24]. Our study displayed PGPIPN can remarkably change the pathologic condition, decrease hepatic MDA level, and increase SOD and GSH-PX levels against alcohol-induced oxidative stress through serum biochemistry assays and liver histopathogical examinations.

ACC and PPAR play vital roles in fat metabolism [25–27]. ACC is a rate limiting enzyme for fatty acid biosynthesis in the liver, which is inactivated by phosphorylation. PPAR regulates transcription of a set of genes containing the peroxisome proliferator response elements, which are involved in the mitochondrial and peroxisomal β-oxidation, transport, and export of fatty acids [28, 29]. Our study showed that PGPIPN mainly reduced the fat synthesis in cells by promoting the phosphorylation of ACC. Furthermore, the peptide could also reduce ACC content, but not to be obvious in hepatocellular carcinoma cell line HepG2. Our results also show that PGPIPN could significantly increase the level of PPAR-γ, thereby enhancing intracellular lipolysis. So PGPIPN can effectively suppress hepatic fat accumulation by regulating PPAR and ACC.

CHOP, an endogenous transcription factor, is an accepted marker of endoplasmic reticulum stress. The CHOP expression is less in the cells under normal circumstances. The endoplasmic reticulum stress upregulates *CHOP* gene transcription and leads to a series of cell stress events [30]. CHOP does not directly induce apoptosis, but inhibits *bcl*-2 expression under overexpression and activates Caspase-3 to initiate apoptosis pathway. The activated Caspase-3 (Cleaved caspase-3) specifically cuts some proteins associated with cell activity and triggers apoptosis cascade reaction resulting in cell apoptosis [31]. Our experiments showed that PGPIPN could decrease CHOP and Caspase-3 levels, and reduce the activation of Caspase-3 in the normal liver LO2 cells and liver tissues of model mice, thereby alleviating endoplasmic reticulum stress and enhancing cell activity. However, they were not obvious in hepatocellular carcinoma HepG2 cells. The cancer and normal cells vary greatly in cell membrane proteins and signaling pathways. Our previous studies indicated that PGPIPN could induce apoptosis of ovarian cancer cells [15, 16]. This experiment showed that CHOP and Caspase-3 levels were very low in steatosis hepatocyte model established with hepatocellular carcinoma cell HepG2.

We reasoned that PGPIPN may regulate cell signaling transduction, change the expression levels and/or activities of ACC, PPAR, CHOP and caspase-3, and then take positive effects in the prevention and cure of AFLD. However, the detailed mechanisms of PGPIPN need to be investigated in the future. In this study, we provided compelling evidence for a unique role of PGPIPN in alleviating chronic alcohol intake-induced hepatic steatosis both *in vitro* and *in vivo*, suggesting that PGPIPN has a significant protective effect for liver against AFLD. Thus, these results raise the possibility that PGPIPN may serve as a novel safe therapy agent in the treatment or prevention of AFLD and its associated complications.

## MATERIALS AND METHODS

### Reagents

The PGPIPN (the purity was confirmed by RP-HPLC to be >99.5%) was provided by Shanghai Sangon Biological Engineering Technology. RPMI 1640 medium and fetal bovine serum (FBS) were purchased from Gibco. Oil Red O and Hematoxylin solution were purchased from Sigma, USA. The WST-1 (water-soluble tetrazolium 1) cell viability and cytotoxicity assay kits were purchased from Beyotime, Haimen, China. TRIzol reagent and Power SYBR Green PCR master mix kit were purchased from Applied Biosystem, Inc., USA. Mouse monoclonal antibodies of human or mouse ACC (catalog #: sc-390344), pACC (catalog #: sc-271965), PPAR-γ (catalog #: sc-271392), CHOP (catalog #: sc-166682) and β-Actin (catalog #: sc-130300) were purchased from Santa Cruz Biotechnology, Inc., USA. Rabbit polyclonal antibodies of human or mouse Caspase-3 (catalog #: 9662) and Cleaved caspase-3 (Asp175) (catalog #: 9661) were purchased from Cell Signaling Technology, Inc., USA. The horseradish peroxidase conjugated secondary antibody (goat anti-mouse or goat anti-rabbit IgG), fluorescent isothiocyanate (FITC)-conjugated secondary antibody (goat anti-mouse IgG), 4′,6-diamidino-2-phenylindole (DAPI) and Super Signal West Pico Trial Kit (enhanced chemiluminescence (ECL) chromogenic reagent kit) were purchased from Pierce, USA.

### Model of steatosis hepatocyte induced by alcohol and pharmacological intervention

Human normal hepatic cell line LO2 (HL-7702) cells were obtained from Shanghai Institutes for Biological Sciences, Chinese Academy of Sciences. Human hepatocellular carcinoma cell line HepG2 cells were originally purchased from ATCC. LO2 and HepG2 cells were cultured in RPMI 1640 with 10% FBS at 37° in 5% CO_2_. Based on the preliminary experiment, we selected 0.5% vol (85.63mmol/L) alcohol as induced dose for LO2 cells, and 0.6% vol (102.76 mmol/L) alcohol for HepG2 cells. LO2 cells and HepG2 cells were respectively divided into five groups in triplicate: control, model, PGPIPN 1, 2 and 3, and seeded respectively into 100 ml cell culture flasks containing 0.5% vol (LO2) or 0.6% vol (HepG2) alcohol, except for control group. PGPIPN 1, 2 and 3 groups respectively contained 0.15, 1.5 and 15μmol/L PGPIPN. Periodic subcultures were carried out for two months. All groups’ cells survived in culture for over 12 passages. Then steatosis hepatocytes induced by alcohol were identified and used for subsequent experiments.

### Assays of biochemical materials related to hepatocyte injury and lipid metabolism in the model of steatosis hepatocyte

The above periodic subcultural LO2 and HepG2 cells were respectively seeded into 24-well plates in sextuplicate at a starting density of 5×10^4^ cells/well in 500μl medium and cultured for 48h. The culture medium composition and conditions of the cells in each group were the same as the above. ALT and AST leaking in the culture medium were determined using Roche Cobas Automatic Biochemical Analyzer (Basel, Switzerland). The cultured cells were digested, collected and lysed. The intracellular TG in 200μl lysate/well was quantified with commercially available kit (Nanjing Jiancheng Technology Co. Ltd., Nanjing, China) according to the manufacture's instruction. Each experiment was performed in three independent sets.

### Morphological observation of liver cell line LO2 and HepG2

The above periodic subcultural LO2 and HepG2 cells were seeded into 6-well cell climbing slice culture plates in sextuplicate at a starting density of 1×10^6^ cells/well in 2ml medium. Cell culture conditions were same as the above. The cells grew to 70–80% confluent for next experiment. Each experiment was performed in three independent sets.

For analysis of fat accumulation in hepatocyte, the cells were stained with Oil Red O and observed by light microscopy. The Oil Red O staining LO2 and HepG2 cells were analyzed with Image-Pro Plus 7.0 image analysis software. The five visual fields were randomly selected in each slice to research liver cell steatosis by quantitative analysis. The fields of the lipid droplets were measured in the total area.

Positive area in measurement area (AA) (%) = positive area/total area×100%

The morphologies of liver cell line LO2 treaded with alcohol and/or peptide were also observed by transmission electron microscope, according to the manufacture's instruction and reference [32] for the processing and operation procedures of cells.

### Cell viability assay of liver cell line LO2 and HepG2

The above periodic subcultural LO2 and HepG2 cells were seeded into 96-well plates in sextuplicate at a starting density of 5 × 10^3^ cells/well and cultured for 48, respectively. The culture medium composition and conditions of the cells in each group were the same as the above. The vitalities of cells were measured with WST-1 method. Treated cells as described above were treated with WST-1 reagent for 4 h at 37°. The cells were rocked on a shaker for 2min, the supernatants were collected and measured at 450nm. The percent viability of cells was calculated using the following formula. Each experiment was performed in three independent sets.

Viability (%) = (the experimental group A_450nm_ value/control group A_450_nm value) × 100%

### Animal model of alcoholic fatty liver disease

Sixty healthy male Kunming mice, 18~22g body weight, were purchased from the Anhui Provincial Center for Medical Experimental Animals (No. 0010394). All animal experiments were carried out under the protocol (No. LLSC20140064) approved by the Institutional Animal Care and Use Committee of Anhui Medical University. All methods and experimental protocols used for relevant studies reported herein, including the use of animals, cell cultures and pertinent *in vivo* studies were carried out in accordance with relevant guidelines and regulations of the protocol of the Committee. All mice were kept in SPF-class (specified pathogen free) sterile room in the Anhui Provincial Center for Medical Experimental Animals. During animal experiments, as far as possible animal sufferings were ameliorated. All mice were fed a nutritionally adequate liquid control diet for a week, and then divided into six groups (control, model, PGPIPN I, PGPIPN II, PGPIPN III and GSH) with 10 mice in each group. In order to make the animal model of AFLD, all mice except control groups were gavaged a single dose of alcohol each day according to the following method: 10ml (5.14mol/L alcohol) /kg body weight in the first 4 weeks, 11ml (6.85mol/L alcohol) /kg body weight in the second 4 weeks, 12ml (8.56mol/L alcohol) /kg body weight in the final 4 weeks. Control group was gavaged the same volume saline. PGPIN I, II and III groups were respectively gavaged PGPIN at 0.025mg, 0.25mg and 2.5mg/kg body weight each day before alcohol gavage, and GSH group was gavaged GSH at 2.5mg/kg body weight as positive control. At the end of the experiment the mice were weighed and anesthetized, serum and liver samples were collected, weighed (liver) and preserved. The liver indexes were calculated.

Liver index (%) = liver weight/body weight

Some portions of liver tissues were fixed in 10% neutral buffered formalin and embedded in paraffin, and the others were snap frozen in liquid nitrogen until use.

### Cell morphological observation of liver tissues and calculation of hepatic steatosis in animal model

Some liver tissues were stained with H&E, with procedure according to reference [33]. These liver tissues were observed and analyzed by light microscopy.

For analysis of fat accumulation in the liver, we observed the liver tissues stained with Oil Red O under high power microscope. Hepatic steatosis was divided into 0-4 degrees according to the proportion of steatosis area by observing Oil Red O staining specimens, 0 for normal liver (<10%), 1 for slight fatty liver (11%-33%), 2 for mild fatty liver (34%-50%), 3 for moderate fatty liver (51%-66%) and 4 for severe fatty liver (>66%). The Oil Red O staining tissue sections were analyzed with Image-Pro Plus 7.0 image analysis software. The two slices from left and right hepatic lobes tissue with good staining were selected to observe under the microscope. The five visual fields were randomly selected in each slice to research liver steatosis by quantitative analysis. The next procedures are the same as above LO2 cells.

### Assays of biochemical materials related to liver injury and lipid metabolism in the serums and liver tissues of animal model

Serum concentrations of TG, TC, LDL-C, HDL-C, ALT and AST were determined using Roche Cobas Automatic Biochemical Analyzer. The liver homogenate of 10% w/v was prepared from the fresh liver tissue and saline. The liver TG was quantified with above commercially available kit.

### Assays of biochemical materials related to liver oxidation in the liver tissues of animal model

The liver homogenate of 10% w/v was prepared from the fresh liver tissue and saline. Total protein, ATP, MDA, SOD and GSH-PX were quantified with commercially available kits (Nanjing Jiancheng Technology Co. Ltd.) according to the manufacture's instruction and reference [34].

### Real time PCR

Total RNA was prepared from cell lines or tissues using TRIzol reagent according to the manufacturer's instructions. Hepatic expressions of *ACC*, *PPAR*-γ, *CHOP* and *caspase*-3 genes were measured by real-time quantitative PCR, using Applied Biosystem Real-time PCR system 7500. Real time PCR adopted TaKaRa SYBR Green as real time PCR Master Mix, which were performed according to the manufacturer's instructions. The relative mRNAs of all samples were calculated using the 2^-ΔΔCt^ method [35]. The β-*actin* was used as a housekeeping gene. The primers synthesized by Shanghai Sangon Biological Engineering Technology are listed in Supplementary Table 1. All reactions were performed in triplicate, and a mixture lacking a complementary DNA template (NTC) was used as the negative control. Each experiment was performed in three independent sets.

### Western blot

Total proteins were extracted from cell lines or tissues, subjected to SDS-PAGE and analyzed by immunoblots as described previously [36, 37]. Primary antibodies were mouse monoclonal antibodies of human and mouse (ACC, pACC, PPAR-γ, CHOP and β-Actin, 1:1000 dilution), and rabbit polyclonal antibodies of human and mouse (Caspase-3 and Cleaved caspase-3, 1:1000 dilution). The sources and catalogs of primary antibodies were listed in above Reagents section. The secondary antibodies conjugated with a horseradish peroxidase included goat anti-mouse and goat anti-rabbit IgG, 1:8000 dilutions. The proteins were detected with ECL system followed by exposure in ChemiScope 6300 Fluorescence and Chemilluminescence Imaging System (Clinx Science Instruments Co., Ltd., Shanghai, China), in which digital images were captured and the intensities were quantified using Quantity-One software version 4.62 (Bio-Rad, USA). The β-Actin was used as a loading control. Each experiment was performed in three independent sets.

### Immunofluorescence staining for detecting PPAR-γ

LO2 cells cultured on NEST chamber slides were fixed with cold methanol at −20°C for 15min. After blocking with 10% normal goat serum in PBS at room temperature for 1h, the cells were incubated with mouse anti-PPAR-γ (primary antibody, 1:1000 dilution) at 4°C for 12h, followed by incubation with FITC-conjugated goat anti-mouse IgG (second antibody, 1:3000 dilution) at 37°C for 60min. DAPI was used to counterstain nucleus. The slides were mounted with ProLong Gold antifade reagent (Thermo, USA) for observation under a fluorescence microscope (IX73, Olympus, Japan). The relative changes of PPAR-γ expression were quantified by ImageJ software (version 1.44, National Institutes of Health, Bethesda, USA).

### Statistical analysis

The results were expressed as the means ± SD. SPSS version 20.0 for Windows was used for all analyses. The statistical significance between experimental groups was determined by analysis of one-way analysis of variance. *P*<0.05 was considered as statistically significant.

## SUPPLEMENTARY MATERIALS FIGURE AND TABLE



## Abbreviations

AFLD: alcoholic fatty liver disease
TC: total cholesterol
TG: total triglyceride
ALT: alanine aminotransferase
AST: asparatate aminotransferase
ACC: acetyl-CoA carboxylase
PPAR-γ: peroxisome proliferator-activated receptor gamma
CHOP: CCAAT/enhancer-binding protein homologous protein
GSH: glutathione
LDL-C: low density lipoprotein-cholesterol
HLD-C: high density lipoprotein-cholesterol
ATP: adenosine triphosphate
MDA: malondialdehyde
SOD: superoxide dismutase
GSH-PX: glutathione peroxidase.
