# Supplementary material for: Therapeutic hexapeptide (PGPIPN) prevents and cures alcoholic fatty liver disease by affecting the expressions of genes related with lipid metabolism and oxidative stress
Source: Oncotarget. 2017 Sep 30;8(50):88079–93. doi: 10.18632/oncotarget.21404 (PMC5675695; doi:10.18632/oncotarget.21404)
Supplement: Supplementary file 1 [file oncotarget-08-88079-s001.pdf]

Therapeutic hexapeptide (PGPIPN) prevents and cures alcoholic fatty liver disease by affecting the expressions of genes related with lipid metabolism and oxidative stress

SUPPLEMENTARY MATERIALS

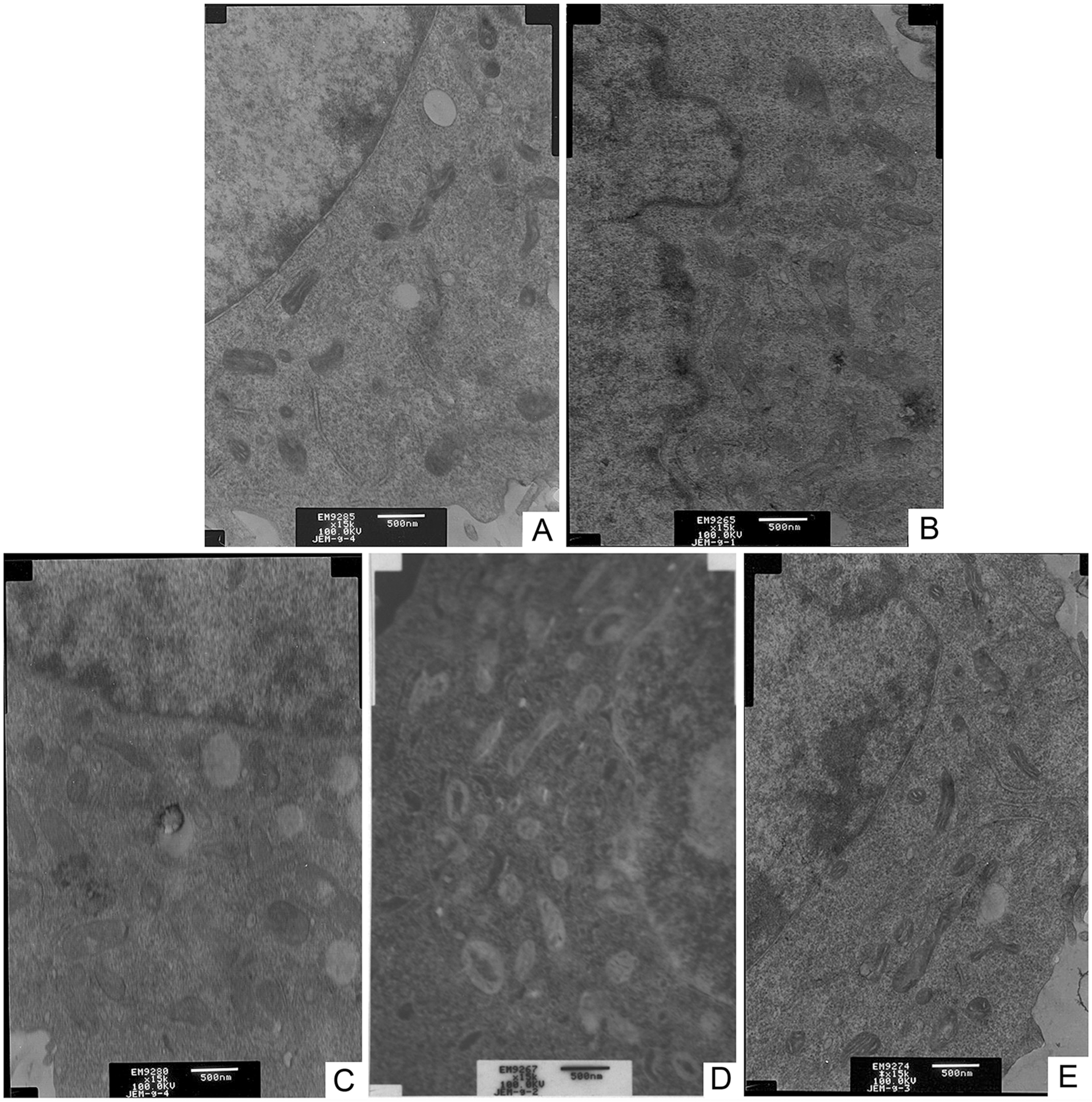

Supplementary Figure 1: PGPIPN alleviated alcohol-induced cell injuries in human liver cell line LO2 (transmission electron microscope observed, ×15000). **(A)** Control group. **(B)** Model group induced by alcohol. **(C)** PGPIPN 1 group treaded with 0.15μmol/L PGPIPN. **(D)** PGPIPN 2 group treaded with 1.5μmol/L PGPIPN. **(E)** PGPIPN 3 group treaded with 15μmol/L PGPIPN.

Supplementary Table 1: The designed primers of *ACC*, *PPAR*-γ, *CHOP* and *caspase*-3 genes in both human and mouse for real time RCR

	Gene	Primer
human	*ACC*	forward 5′-CAAGCCGATCACCAAGAGTAAA-3′
		reverse 5′-CCCTGAGTTATCAGAGGCTGG-3′;
	PPAR-γ	forward 5′-TACTGTCGGTTTCAGAAATGCC-3′
		reverse 5′-GTCAGCGGACTCTGGATTCAG-3′
	CHOP	forward 5′-GGAAACAGAGTGGTCATTCCC-3′
		reverse 5′-CTGCTTGAGCCGTTCATTCTC-3′
	caspase-3	forward 5′-CATGGAAGCGAATCAATGGACT-3′
		reverse 5′-CTGTACCAGACCGAGATGTCA-3′
	β-*actin*	forward 5′-CATGTACGTTGCTATCCAGGC-3′
		reverse 5′-CTCCTTAATGTCACGCACGAT-3′
mouse	*ACC*	forward 5′-GATGAACCATCTCCGTTGGC-3′
		reverse 5′-GACCCAATTATGAATCGGGAGTG-3′
	PPAR-γ	forward 5′-TCGCTGATGCACTGCCTATG-3′
		reverse 5′-GAGAGGTCCACAGAGCTGATT-3′
	CHOP	forward 5′-CTGGAAGCCTGGTATGAGGAT-3′
		reverse 5′-CAGGGTCAAGAGTAGTGAAGGT-3
	caspase-3	forward 5′-ATGGAGAACAACAAAACCTCAGT-3′
		reverse 5′-TTGCTCCCATGTATGGTCTTTAC-3′
	β-*actin*	forward 5′-GAAATCGTGCGTGACATCAAAG-3′
		reverse 5′-TGTAGTTTCATGGATGCCACAG-3′
